# Complete genome sequence of *Pseudoalteromonas* phage vB_PspS-H40/1 (formerly H40/1) that infects *Pseudoalteromonas* sp. strain H40 and is used as biological tracer in hydrological transport studies

**DOI:** 10.1186/s40793-017-0235-5

**Published:** 2017-02-02

**Authors:** René Kallies, Bärbel Kiesel, Matthias Schmidt, Johannes Kacza, Nawras Ghanem, Anja Narr, Jakob Zopfi, Lukas Y. Wick, Jörg Hackermüller, Hauke Harms, Antonis Chatzinotas

**Affiliations:** 1 Department of Environmental Microbiology, Helmholtz Centre for Environmental Research - UFZ, 04318 Leipzig, Germany; 2 Department of Isotope Biogeochemistry, ProVis - Centre for Chemical Microscopy, Helmholtz Centre for Environmental Research - UFZ, 04318 Leipzig, Germany; 3 Institute of Anatomy, Histology and Embryology, Faculty of Veterinary Medicine, University of Leipzig, 04103 Leipzig, Germany; 4 Department of Environmental Sciences - Aquatic and Stable Isotope Biogeochemistry, University of Basel, 4056 Basel, Switzerland; 5 Young Investigators Group Bioinformatics & Transcriptomics, Helmholtz Centre for Environmental Research - UFZ, 04318 Leipzig, Germany; 6 German Centre for Integrative Biodiversity Research (iDiv) Halle-Jena-Leipzig, Deutscher Platz 5e, 04103 Leipzig, Germany

**Keywords:** *Pseudoalteromonas* phage, *Siphoviridae*, AquaDiva, Marine phage, Virus, Genome, Bacteriophages as tracers

## Abstract

*Pseudoalteromonas phage* vB_PspS-H40/1 is a lytic phage that infects *Pseudoalteromonas* sp. strain H40. Both, the phage and its host were isolated in the 1970s from seawater samples collected from the North Sea near the island of Helgoland, Germany. The phage particle has an icosahedral capsid with a diameter of ~43 to 45 nm and a long non-contractile tail of ~68 nm in length, a typical morphology for members of the *Siphoviridae* family. The linear dsDNA genome of *Pseudoalteromonas* phage vB_PspS-H40/1 has a sequence length of 45,306 bp and a GC content of 40.6%. The genome has a modular structure and contains a high proportion of sequence information for hypothetical proteins, typically seen in phage genome sequences. This is the first report of the complete genome sequence of this lytic phage, which has been frequently used since the 1990s as biological tracer in hydrogeological transport studies.

**Electronic supplementary material:**

The online version of this article (doi:10.1186/s40793-017-0235-5) contains supplementary material, which is available to authorized users.

## Introduction

*Pseudoalteromonas* *,* affiliated with the order *Alteromonadales* [1, 2] of the *Gammaproteobacteria* [2, 3]*,* is a genus of heterotrophic, Gram-negative marine bacteria [4]. Members of this genus are widely distributed in marine ecosystems and have attracted interest due to their frequent association with eukaryotic hosts and their production of biologically active compounds [5–7]. Both inhibitory as well as synergistic chemical interactions between strains of *Pseudoalteromonas* and various marine eukaryotes have been described [8], indicating that members of this genus are potentially involved in complex ecological networks across trophic levels. Viruses, as the most abundant biological entity in the oceans, are a major cause of host mortality and thus key players within these ecological networks; they influence host community structures and thereby also influence global biogeochemical cycles and genetic landscapes [9].

As of April 2016, 14 complete *Pseudoalteromonas* phage genomes have been deposited at GenBank (10 of them unpublished). Ten representatives belong to the *Caudovirales* order (three siphoviruses, four podoviruses, two myoviruses and one unclassified caudovirus), one is a corticovirus and three are unclassified viruses. *Pseudoalteromonas* phages have been shown to represent a significant group of phages in the ocean [10, 11], making it likely that the number of yet unknown phage genomes is much higher. Characterization of additional *Pseudoalteromonas* phage genomes is a further step towards a better understanding of the diversity, the biology and the ecological impact of this group of phages and contributes to an improved interpretation of viral metagenome data and dynamics of viral populations in the environment [12–14]. Moreover, comparison of potentially closely related viral genomes is a prerequisite to understand virus evolution and intraspecies genomic variation [15, 16].

In this report we describe the genome of the *Pseudoalteromonas* phage vB_PspS-H40/1, isolated in 1978 from the North Sea near the island of Helgoland (Germany) [17]. Notably, this phage has been used as a non-reactive biological agent to trace the flow of water in surface and subsurface environments and promises utility in (geo-)hydrological transport studies [18–21]. According to the scheme for the nomenclature of viruses the phage was re-named from H40/1 to vB_PspS-H40/1 [22].

## Organism Information

### Classification and features

The bacterial host H40 was isolated from seawater samples collected between 1969 and 1978 near the island of Helgoland in the North Sea [17]. Sequence analysis of the 16S-rRNA gene revealed H40 as a member of the *Pseudoalteromonas* genus. The partial 16S-rRNA sequence was deposited at GenBank (acc. no. KX236488). Strain H40 was used as the bacterial host for screening of lytic marine bacteriophages from the same sampling site resulting in the isolation of phage vB_PspS-H40/1 [17].

**Fig. 1:**
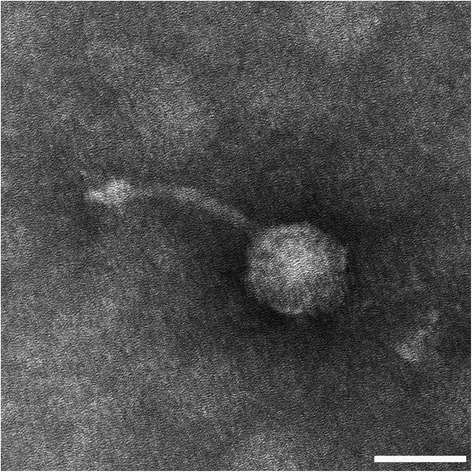
Transmission electron micrograph of *Pseudoalteromonas* phage vB_PspS-H40/1 infecting *Pseudoalteromonas* sp. strain H40. Virus particles were stained with 2% tungstophosphoric acid and visualized using an electron microscope Libra 120 (Zeiss, Oberkochen, Germany). Size bar: 40 nm

*Pseudoalteromonas* phage vB_PspS-H40/1 is a lytic phage forming clear, well-contrasted plaques of four to five mm in diameter. Transmission electron microscopy showed that vB_PspS-H40/1 is a B1 morphotype with an icosahedral capsid of 42.7 nm in length (±1.7 nm) and 44.5 nm in width (± 2 nm). The long, non-contractile tail had a length of 67.5 nm (± 3.9 nm) and a diameter of 6.7 nm (± 0.6 nm) (Fig. 1). These morphological characteristics are typical for members belonging to the *Siphoviridae* family of the order *Caudovirales* [23].

The phage surface is moderately charged (zeta potential of −11 ± 3 mV (100 mM K_2_HPO_4_/KH_2_PO_4_, pH = 7)) and of moderate hydrophobicity (water contact angle = 53 ± 3°) as determined by standard physico-chemical characterization methods of bacterial surfaces (e.g. [24]).

**Fig. 2:**
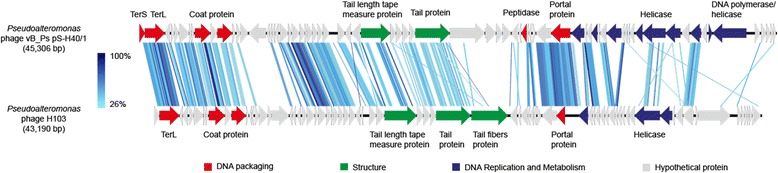
Genome maps of *Pseudoalteromonas* phages vB_PspS-H40/1 and H103. Protein coding sequences are presented by arrows and their functions are indicated by colours: red, DNA packaging; green, structural genes, blue, DNA replication and metabolism; grey, hypothetical proteins. Similarities between both genomes were calculated using tblastx [36]. Similarities are shown in blue according to the scale on the left side. The figure was drawn using Easyfig [44]

**Fig. 3:**
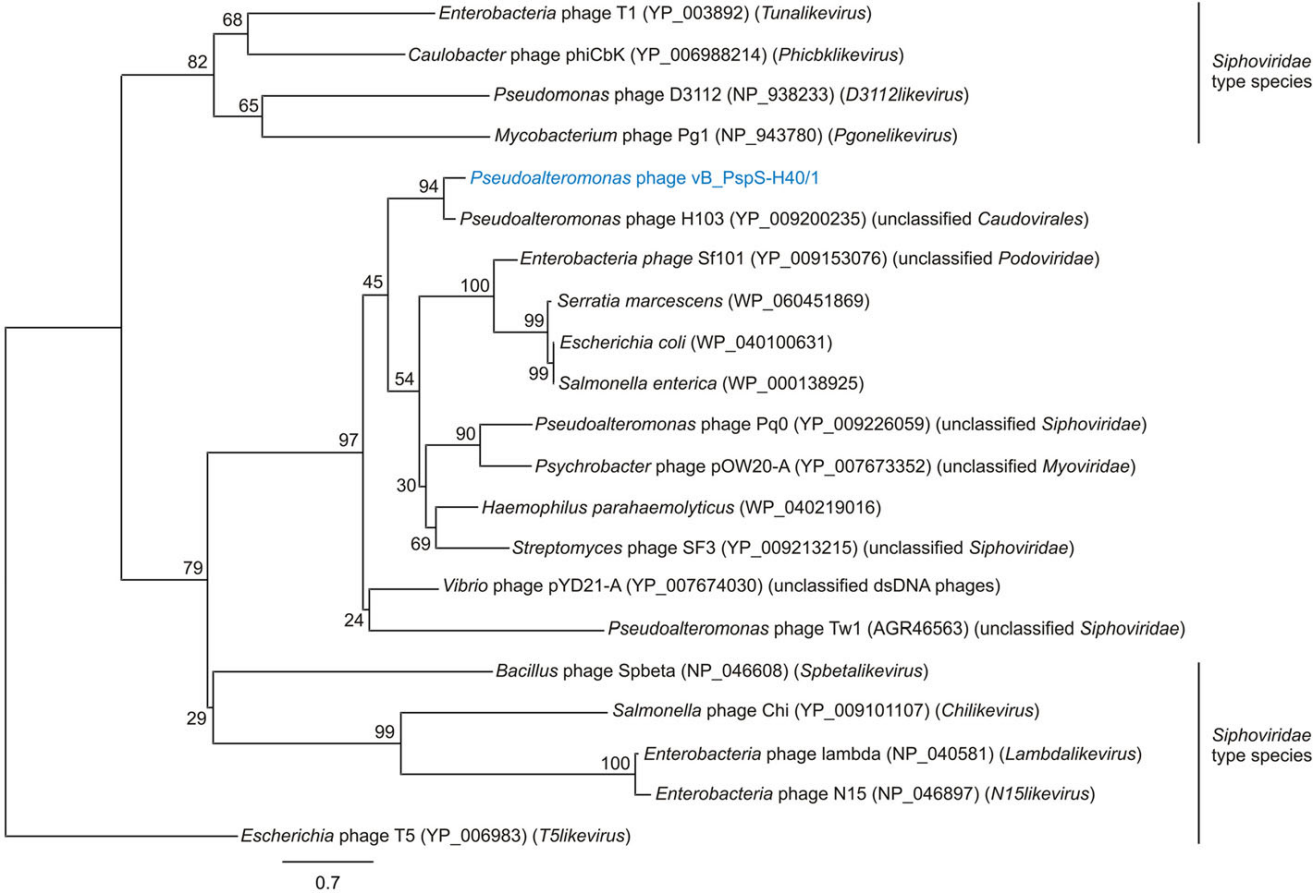
Maximum-likelihood phylogenetic tree based on the TerL amino acid sequences indicating the phylogenetic relationship of *Pseudoalteromonas* phage vB_PspS-H40/1 (shown in blue) to related phages and bacterial sequences (probably prophages). Analyses were performed guided by the Jones-Taylor-Thornton substitution model using PhyML [45]. Confidence testing was performed by 500 bootstrap replicates. Bootstrap values are shown next to the nodes. GenBank accession numbers and genera are shown in parentheses. Bar represents 0.7 substitution per amino acid position

*Pseudoalteromonas* phage vB_PspS-H40/1 has a linear dsDNA genome comprising 45,306 bp with a GC content of 40.6%. It showed the highest similarity (55.3% identity) over the whole genome to *Pseudoalteromonas* phage H103 (GenBank acc. no. KP994596), an unclassified representative of the *Caudovirales* order infecting the marine host *Pseudoalteromonas marina* [25] (Fig. 2). Phylogenetic analysis of the terminase large subunit (TerL) amino acid sequence grouped phage vB_PspS-H40/1 together with phage H103 in one clade (Fig. 3). Both phages shared a most recent common ancestor with TerL sequences found in unclassified members of the *Caudovirales* order and (probably) prophage sequences from members of the bacterial family *Enterobacteriaceae* [26, 27]. These unclassified phages belong to all three families of the *Caudovirales* order, i.e. *Siphoviridae*, *Podoviridae* and *Myoviridae*. Taken together, TerL-based phylogenetic analysis indicates phage vB_PspS-H40/1 occupies (perhaps together with phage H103) a phylogenetic position distinct from established genera of the *Siphoviridae* family.

Phylogenetic classification and general features of *Pseudoalteromonas* phage vB_PspS-H40/1 are summarized in Table 1.

**Table 1:** Classification and general features of *Pseudoalteromonas* phage vB_PspS-H40/1 infecting *Pseudoalteromonas* sp. strain H40

MIGS ID	Property	Term	Evidence code^a^
	Classification	Domain: N/A	
		Genome group: dsDNA viruses, no RNA stage	IDA
		Phylum: unassigned	
		Class: unassigned	
		Order: *Caudovirales*	TAS [23]
		Family: *Siphoviridae*	TAS [23]
		Genus: unassigned	
		Species: unassigned	
		Strain: vB_PspS-H40/1	
	Particle shape	Isometric capsid, long non-contractile tail	IDA
	Gram strain	N/A	
	Cell shape	N/A	
	Motility	N/A	
	Sporulation	N/A	
	Temperature range	N/A	
	Optimum temperature	N/A	
	pH range; optimum	N/A	
	Carbon source	N/A	
MIGS-6	Habitat	Marine water column	TAS [17]
MIGS-6.3	Salinity	N/A	
MIGS-22	Oxygen requirement	N/A	
MIGS-15	Biotic relationship	Intracellular parasite of *Pseudoalteromonas* sp. strain H40	TAS [17]
MIGS-14	Pathogenicity	Virulent phage of *Pseudoalteromonas* sp. strain H40	IDA
MIGS-4	Geographic location	North Sea, Helgoland, Germany	TAS [17]
MIGS-5	Sample collection	1978	TAS [17]
MIGS-4.1	Latitude	54°10'N	IDA
MIGS-4.2	Longitude	7°52'E	IDA
MIGS-4.4	Altitude	N/A	

^a^Evidence codes - *IDA* inferred from direct assay, *TAS* traceable author statement, *N/A* not applicable. These evidence codes are from the Gene Ontology project [46]

## Genome sequencing information

### Genome project history

*Pseudoalteromonas* phage vB_PspS-H40/1 is one of the few known marine siphovirus isolates [28] and belongs to a group of important phages found in the ocean [10, 11]. Genome sequencing of this phage will increase available information and facilitate future studies on diversity, evolution and ecological impact of marine viruses. A second reason to select this phage for sequencing is its frequent application in biological tracing experiments [18–21]. Phage vB_PspS-H40/1 is one of the marine phages that are currently used in the frame of the Collaborative Research Centre AquaDiva to trace the hydrological flow and reactive transport of colloidal particles from the surface into the Earth’s subsurface [29]. Environmental influences might inactivate a still to define percentage of transported phages. Knowledge of a phage genome will facilitate the detection of this phage using PCR and thus allow to (quantitatively) distinguish between biologically active (e.g. detected by plaque assay) from inactive phages and might hence help in the interpretation of findings from these transport experiments.

The dsDNA genome of phage vB_PspS-H40/1 was sequenced using the Illumina MiSeq system. Experiments, genome assembly, annotation and submission to GenBank were performed at the Department of Environmental Microbiology at the Helmholtz Centre for Environmental Research - UFZ, Leipzig, Germany. The sequencing project was started in December 2015 and finished in February 2016 and its outcome is available in the Genome Online Database under project number Gp0133998. The complete annotated genome sequence was submitted to Genbank (GenBank acc. no. KU747973). Information on the project is summarized in Table 2.

**Table 2:** Project information

MIGS ID	Property	Term
MIGS 31	Finishing quality	Finished
MIGS-28	Libraries used	One paired-end Illumina library
MIGS 29	Sequencing platforms	Illumina MiSeq
MIGS 31.2	Fold coverage	~1200x
MIGS 30	Assemblers	Geneious Assembler version R6
MIGS 32	Gene calling method	RAST, GLIMMER, GeneMark.hmm
	Locus Tag	NA^a^
	Genbank ID	KU747973
	GenBank Date of Release	Jun 07, 2016
	GOLD ID	Gp0133998
	BIOPROJECT	NA^a^
MIGS 13	Source Material Identifier	NA^a^
	Project relevance	Diversity of marine bacteriophage, Hydrological transport studies

^a^ *NA* not available

### Growth conditions and genomic DNA preparation

The bacterial host *Pseudoalteromonas* sp., strain H40 was grown and maintained in 2216E medium [30] (containing nutrients at 50% of the original concentration) at 20 °C. The phage was propagated on its host in petri dishes with 2216E agar (with nutrients as above) using the double agar-layer technique. Five ml of SM buffer (100 mM NaCl, 8 mM MgSO_4_ × 7H_2_O, 50 mM Tris–HCl, pH 7.5) and a few drops of chloroform were added to the plates after confluent lysis of bacterial host cells. Plates were gently shaken for 2 h at room temperature, supernatant was collected and cell debris was removed by centrifugation at 10,000 × g for 15 min. One volume of chloroform was then added to the supernatant, gently mixed and centrifuged at 5,000 × g for 5 min. The phage particle-containing upper phase was passed through a 0.22 μm polyvinylidene fluoride CHROMAFIL® membrane filter to remove unlysed host cells and debris. The resulting phage suspension was stored at 4 °C. DNA from phage particles was extracted following the protocol of Thurber et al. [31].

### Genome sequencing and assembly

The extracted phage DNA was sheared into ~300 to 500 bp fragments using the Covaris M220 Focused-ultrasonicator™ instrument and one paired-end library was prepared with the NEBNext® Ultra™ DNA Library Prep Kit for Illumina®. Sequencing was performed at the Helmholtz Centre for Environmental Research - UFZ on an Illumina MiSeq system (2 × 150 bp). In total, 418,468 paired-reads were obtained for *Pseudoalteromonas* phage vB_PspS-H40/1. Raw reads were split into 10 subsets (approximately 42,000 reads for each subset) to facilitate improved assembly [32]. Independent assemblies were performed for each subset by Geneious Assembler (version R6) resulting in nearly the same single contig for each of the subsets but with different starting points indicating a circularly permuted genome of phage vB_PspS-H40/1. For confirmation, PCR primers were designed matching the ends of the contigs with an outward orientation and used in PCR. The resulting amplicon was Sanger sequenced and used to close the contigs for *Pseudoalteromonas* phage vB_PspS-H40/1. The coverage was estimated by reference mapping of the raw reads to the contig resulting in an approximate 1200-fold coverage (~ 92% of all reads) of the 45,306 bp genome.

### Genome annotation

Genes and ORFs in the phage genome were predicted using a combination of three gene calling methods: the RAST annotation server [33], GLIMMER3 [34] and GeneMark.hmm [35]. Only ORFs that were predicted by two of the three gene calling methods were included in the annotation. Functional annotation of translated ORFs was improved by BLASTp alignments against the NCBI non-redundant database [36]. In addition, RPS-BLAST searches against the Conserved Domain Database [37] and HMMER search [38] against the UniProtKB database were performed. Protein domains were predicted using the COG [39], Pfam [40], TIGRFAMs [41] and KEGG [42] databases. Phoebius [43] was used to predict signal peptides and transmembrane helices.

## Genome properties

The complete genome of *Pseudoalteromonas* phage vB_PspS-H40/1 was assembled into one linear contig of 45,306 bp with a GC content of 40.6%. In total, 73 putative coding sequences were predicted in the phage genome (Fig. 2, Additional file 1: Table S1). Seventeen of these 73 protein coding genes were assigned to putative protein functions. The functions of the remaining 56 putative protein coding genes remained unknown and they were annotated as hypothetical proteins. One gene with a signal peptide was identified together with eight proteins containing transmembrane helices. *Pseudoalteromonas* phage vB_PspS-H40/1 genome properties are summarized in Table 3 and genes assigned to COG functional categories are listed in Table 4.

**Table 3:** Genome statistics

Attribute	Value	% of Total
Genome size (bp)	45,306	100.00
DNA coding (bp)	42,786	94.44
DNA G + C (bp)	17,376	40.60
DNA scaffolds	1	100.00
Total genes	73	100.00
Protein coding genes	73	100.00
RNA genes	0	0.00
Pseudo genes	0	0.00
Genes in internal clusters	0	0.00
Genes with function prediction	17	23.29
Genes assigned to COGs	6	8.22
Genes with Pfam domains	18	24.66
Genes with signal peptides	1	1.34
Genes with transmembrane helices	8	10.96
CRISPR repeats	0	0.00

**Table 4:** Number of genes associated with general COG functional categories

Code	Value	%age	Description
J	0	0.00	Translation, ribosomal structure and biogenesis
A	0	0.00	RNA processing and modification
K	1	1.34	Transcription
L	3	4.11	Replication, recombination and repair
B	0	0.00	Chromatin structure and dynamics
D	0	0.00	Cell cycle control, Cell division, chromosome partitioning
V	0	0.00	Defense mechanisms
T	0	0.00	Signal transduction mechanisms
M	1	1.34	Cell wall/membrane biogenesis
N	0	0.00	Cell motility
U	0	0.00	Intracellular trafficking and secretion
O	0	0.00	Posttranslational modification, protein turnover, chaperones
C	0	0.00	Energy production and conversion
G	0	0.00	Carbohydrate transport and metabolism
E	0	0.00	Amino acid transport and metabolism
F	0	0.00	Nucleotide transport and metabolism
H	0	0.00	Coenzyme transport and metabolism
I	0	0.00	Lipid transport and metabolism
P	0	0.00	Inorganic ion transport and metabolism
Q	0	0.00	Secondary metabolites biosynthesis, transport and catabolism
R	1	1.34	General function prediction only
S	1	1.34	Function unknown
-	66	90.41	Not in COGs

The total is based on the total number of protein coding genes in the genome

## Insights from the genome sequence

When all 73 predicted CDSs were subjected to functional annotation only 17 CDSs could be assigned to a specific function. These functions were related to DNA packaging, head and tail assembly, DNA replication and metabolism (Fig. 2 and Additional file 1: Table S1). Twenty-nine of the predicted CDSs, including mainly hypothetical proteins but also TerL and structural proteins, showed highest similarity to the unclassified *Caudovirales* member *Pseudoalteromonas* phage H103 after blastp analysis (Fig. 2). Highest similarity of other CDSs was found to marine *Pseudoalteromonas* phages belonging to the *Siphoviridae* family, i.e. *Pseudoalteromonas* phage TW1 (GenBank acc. no. KC542353), *Pseudoalteromonas* phage Pq0 (GenBank acc. no. NC_029100) and *Pseudoalteromonas* phage H105/1 (GenBank acc. no. NC_015293). However, proteins involved in DNA replication (helicase, RecA-NTPase and methylase) were related to those found in *Vibrio* phage H188 (GenBank acc.no. KT160311) and *Escherichia* phage vB_EcoM-ep3 (GenBank acc. no. NC_025430), two members of the *Myoviridae* family, suggesting mosaicism of the genome.

Phylogenetic inferences deduced from the TerL amino acid sequence showed no close phylogenetic relationship to any of the established *Siphoviridae* genera (Fig. 3).

## Conclusions

The characterized complete genome of lytic *Pseudoalteromonas* phage vB_PspS-H40/1 that was isolated from seawater in the North Sea improves our knowledge of this significant group of phages. The linear dsDNA genome has a size of 45,306 bp and a GC content of 40.6%. The obtained sequencing data indicate phage vB_PspS-H40/1 uses headful packaging strategy and that the genome is circularly permuted. Among the 73 protein coding sequences only 17 were functionally annotated. Transmission electron microscopy and phylogenetic analysis of TerL sequences suggest this phage might belong to a genus of a yet unclassified group of *Siphoviridae*. Next to studies on specific phage-host interactions in marine systems, phage vB_PspS-H40/1 will be used in surface and groundwater tracer experiments and its genome sequence and morphological description will help interpreting results from these studies.

## Additional file

Additional file 1: Table S1. Putative functions of orfs found in *Pseudoalteromonas* phage vB_PS-H40/1 genome. Also shown are most significant blastp hits for each orf. (DOCX 19 kb)

## Abbreviations

TerL: Terminase large subunit
