# Supplementary material for: Complete genome sequence of Pseudoalteromonas phage vB_PspS-H40/1 (formerly H40/1) that infects Pseudoalteromonas sp. strain H40 and is used as biological tracer in hydrological transport studies
Source: Stand Genomic Sci. 2017 Feb 2;12:20. doi: 10.1186/s40793-017-0235-5 (PMC5288847; doi:10.1186/s40793-017-0235-5)
Supplement: Additional file 1: Table S1. — Putative functions of orfs found in Pseudoalteromonas phage vB_PS-H40/1 genome. Also shown are most significant blastp hits for each orf. (DOCX 19 kb) [file 40793_2017_235_MOESM1_ESM.docx]

| **Additional file 1: Table S1.** *Pseudoalteromonas* phage vB_PspS-H40/1 gene annotations. | | | | | | |
| --- | --- | --- | --- | --- | --- | --- |
| **ORF No**. | **Protein size (aa)** | **Strand**  **orientation** | **Putative function** | **Most significant blastp hit (organism)** | **E-value** | **aa identity** |
| 1 | 167 | + | hypothetical protein | hypothetical protein (*Providencia rettgeri*) | 1e-16 | 35% |
| 2 | 137 | + | hypothetical protein | hypothetical protein (*Pseudoalteromonas* phage H103) | 6e-30 | 43% |
| 3 | 821 | + | putative tail protein | tail domain-containing protein (*Pseudoalteromonas* phage TW1) | 3e-116 | 33% |
| 4 | 833 | + | hypothetical protein | hypothetical protein UT24_C0054G0001, partial (*Candidatus Woesebacteria* bacterium GW2011_GWB1_39_12) | 0.018 | 52% |
| 5 | 265 | + | hypothetical protein | hypothetical protein (*Paraburkholderia bannensis*) | 0.003 | 39% |
| 6 | 147 | + | hypothetical protein | hypothetical protein (*Pseudoalteromonas shioyasakiensis*) | 1e-28 | 60% |
| 7 | 200 | + | hypothetical protein | hypothetical protein (*Pseudomonas aeruginosa*) | 2e-05 | 29% |
| 8 | 160 | - | hypothetical protein | hypothetical protein (*Pseudoalteromonas* phage Pq0) | 3e-95 | 91% |
| 9 | 58 | - | hypothetical protein | hypothetical protein (*Pseudoalteromonas* phage Pq0) | 8e-15 | 64% |
| 10 | 130 | - | putative peptidase | hypothetical protein UR61_C0047G0004 (candidate division WS6 bacterium GW2011_GWE1_34_7) | 2e-36 | 50% |
| 11 | 50 | - | hypothetical protein | hypothetical protein (*Pseudoalteromonas* phage H103) | 2e-14 | 68% |
| 12 | 62 | - | hypothetical protein | hypothetical protein (*Pseudoalteromonas* phage H103) | 7e-23 | 71% |
| 13 | 69 | + | hypothetical protein | hypothetical protein (*Vibrio* phage VH7D) | 0.066 | 40% |
| 14 | 357 | - | hypothetical protein | hypothetical protein (*Pseudoalteromonas* phage H103) | 0.0 | 81% |
| 15 | 464 | - | putative portal protein | putative portal protein (*Pseudoalteromonas* phage TW1) | 1e-178 | 58% |
| 16 | 311 | - | putative DNA methylase | DNA methylase (*Escherichia* phage vB_EcoM-ep3) | 2e-129 | 61% |
| 17 | 62 | - | hypothetical protein | hypothetical protein (*Pseudoalteromonas* phage H103) | 5e-07 | 64% |
| 18 | 112 | - | hypothetical protein | hypothetical protein (*Pseudoalteromonas* phage H103) | 6e-57 | 73% |
| 19 | 140 | - | putative DNA-binding protein | hypothetical protein VPNG_00030 (*Vibrio* phage VBP47) | 6e-27 | 43% |
| 20 | 53 | - | hypothetical protein | hypothetical protein TW1_029 (*Pseudoalteromonas* phage TW1) | 2e-10 | 53% |
| 21 | 153 | - | hypothetical protein | hypothetical protein (*Pseudoalteromonas* phage H103) | 1e-76 | 71% |
| 22 | 355 | - | putative DNA-methyltransferase | putative site-specific DNA-methylase (*Enterobacteria* phage ECGD1) | 1e-111 | 53% |
| 23 | 74 | - | hypothetical protein | hypothetical protein (*Pseudoalteromonas* phage H103) | 8e-30 | 69% |
| 24 | 66 | - | hypothetical protein | n.s.s.f. |  |  |
| 25 | 55 | - | hypothetical protein | hypothetical protein LPPPVgp11 (*Listonella* phage phiHSIC) | 1e-12 | 57% |
| 26 | 99 | - | hypothetical protein | n.s.s.f. |  |  |
| 27 | 168 | - | putative RNaseH-like domain protein | putative RNaseH-like domain protein (*Vibrio* phage H188) | 6e-35 | 48% |
| 28 | 544 | - | putative DNA helicase | DNA helicase (*Vibrio* phage H188) | 3e-179 | 50% |
| 29 | 358 | - | putative cas4-like nuclease | YqaJ-like viral recombinase domain-containing protein (*Endozoicomonas montiporae* CL-33) | 2e-103 | 48% |
| 30 | 177 | - | hypothetical protein | hypothetical protein (*Vibrio* phage H188) | 1e-47 | 54% |
| 31 | 260 | - | putative NTPase | putative RecA-like NTPase (*Vibrio* phage H188) | 6e-117 | 64% |
| 32 | 130 | - | hypothetical protein | APHP domain-containing protein (*Streptomyces bambergiensis*) | 2.6 | 31% |
| 33 | 79 | + | putative transcriptional regulator | hypothetical protein (*Delftia acidovorans*) | 2e-05 | 34% |
| 34 | 797 | - | putative bifunctional DNA primase/polymerase | bifunctional DNA primase/polymerase domain protein (*Vibrio* phage H188) | 4e-169 | 38% |
| 35 | 118 | + | hypothetical protein | XRE family transcriptional regulator (*Acidovorax* sp. Root217) | 1.3 | 36% |
| 36 | 124 | + | hypothetical protein | hypothetical protein (*Yersinia enterocolitica*) | 6e-09 | 34% |
| 37 | 100 | + | hypothetical protein | PREDICTED: xin actin-binding repeat-containing protein 1 isoform X2 (*Macaca fascicularis*) | 4.8 | 30% |
| 38 | 65 | + | hypothetical protein | MULTISPECIES: hypothetical protein (*Corynebacterium*) | 9.3 | 33% |
| 39 | 60 | + | hypothetical protein | esterase (*Photobacterium damselae*) | 7.6 | 36% |
| 40 | 130 | + | putative terminase small subunit | hypothetical protein (*Vitreoscilla* sp. SN6) | 4e-36 | 58% |
| 41 | 477 | + | putative terminase large subunit | Terminase large subunit (*Pseudoalteromonas* phage H103) | 0.0 | 82% |
| 42 | 112 | - | hypothetical protein | hypothetical protein (*Pseudoalteromonas* phage H103) | 9e-12 | 40% |
| 43 | 76 | + | hypothetical protein | hypothetical protein AV949_gp40 (*Pseudoalteromonas* phage H105/1) | 1e-32 | 79% |
| 44 | 107 | + | hypothetical protein | hypothetical protein (*Pseudoalteromonas* phage H103) | 6e-39 | 63% |
| 45 | 248 | + | hypothetical protein | hypothetical protein (Pseudoalteromonas phage H103) | 1e-84 | 56% |
| 46 | 59 | + | hypothetical protein | hypothetical protein (*Pseudoalteromonas* phage H103) | 1e-19 | 66% |
| 47 | 58 | + | hypothetical protein | hypothetical protein AV949_gp17 (*Pseudoalteromonas* phage H105/1) | 3e-14 | 67% |
| 48 | 375 | + | putative coat protein | coat protein (*Pseudoalteromonas* phage H103) | 2e-117 | 55% |
| 49 | 149 | + | hypothetical protein | hypothetical protein TW1_056 (*Pseudoalteromonas* phage TW1) | 4e-71 | 77% |
| 50 | 355 | + | putative coat protein | putative coat protein (*Pseudoalteromonas* phage TW1) | 0.0 | 70% |
| 51 | 64 | + | hypothetical protein | n.s.s.f. |  |  |
| 52 | 93 | + | hypothetical protein | hypothetical protein (*Pseudoalteromonas atlantica*) | 1e-11 | 43% |
| 53 | 179 | + | hypothetical protein | n.s.s.f. |  |  |
| 54 | 410 | - | hypothetical protein | hypothetical protein (*Pseudomonas stutzeri*) | 1e-137 | 51% |
| 55 | 57 | - | hypothetical protein | n.s.s.f. |  |  |
| 56 | 63 | - | hypothetical protein | hypothetical protein (*Pseudoalteromonas* phage H103) | 7e-19 | 68% |
| 57 | 68 | - | hypothetical protein | hypothetical protein (*Stenotrophomonas* phage vB_SmaS-DLP_6) | 1.1 | 36% |
| 58 | 84 | - | hypothetical protein | hypothetical protein M316_0070 (*Nitrincola* phage 1M3-16) | 1.8 | 28% |
| 59 | 197 | + | hypothetical protein | hypothetical protein (*Pseudoalteromonas* phage H103) | 2e-56 | 51% |
| 60 | 71 | + | hypothetical protein | hypothetical protein (*Pseudomonas* sp. MT-1) | 1e-04 | 36% |
| 61 | 184 | + | hypothetical protein | hypothetical protein (*Pseudoalteromonas* phage H103) | 9e-77 | 56% |
| 62 | 122 | + | hypothetical protein | hypothetical protein (*Pseudoalteromonas* phage H103) | 3e-62 | 78% |
| 63 | 56 | + | hypothetical protein | n.s.s.f. |  |  |
| 64 | 127 | + | hypothetical protein | hypothetical protein (*Pseudoalteromonas* phage H103) | 2e-58 | 72% |
| 65 | 118 | + | hypothetical protein | hypothetical protein (*Pseudoalteromonas* phage H103) | 3e-53 | 71% |
| 66 | 131 | + | hypothetical protein | hypothetical protein (*Pseudoalteromonas* phage H103) | 1e-69 | 78% |
| 67 | 149 | + | hypothetical protein | hypothetical protein (*Pseudoalteromonas* phage H103) | 4e-67 | 69% |
| 68 | 170 | + | hypothetical protein | hypothetical protein (*Pseudoalteromonas* phage H103) | 2e-41 | 42% |
| 69 | 138 | - | hypothetical protein | hypothetical protein (*Bacillus oceanisediminis*) | 3.4 | 30% |
| 70 | 206 | - | hypothetical protein | hypothetical protein (*Pseudoalteromonas* phage H103) | 2e-30 | 39% |
| 71 | 695 | + | putative tail length tape-measure protein | tail length tape-measure protein 1 (*Pseudoalteromonas* phage H103) | 2e-99 | 36% |
| 72 | 133 | + | hypothetical protein | hypothetical protein (*Yokenella regensburgei*) | 0.018 | 28% |
| 73 | 148 | + | hypothetical protein | hypothetical protein (*Pseudoalteromonas* phage H103) | 0.30 | 27% |
| n.s.s.f: no significant similarity found | | | | | | |
